# Microsurgical resection of a medullary cavernous malformation through a far lateral approach

**DOI:** 10.3171/2019.7.FocusVid.19180

**Published:** 2019-07-01

**Authors:** Santiago Gomez-Paz, Kimberly P. Kicielinski, Ajith Thomas, Christopher S. Ogilvy

**Affiliations:** Department of Neurosurgery, Beth Israel Deaconess Medical Center, Harvard Medical School, Boston, Massachusetts

**Keywords:** microsurgery, medulla, cavernous malformation, posterior fossa, far lateral approach, brainstem, cavernoma, vascular malformation, video

## Abstract

The decision to resect a cavernous malformation of the brainstem is based on patient- and lesion-specific factors. The patient’s age, comorbidities, neurologic condition, and number and severity of symptomatic hemorrhages are crucial to consider.^1,3,5^ The proximity to the brainstem surface, amount of hematoma, and true lesion size help dictate the surgical corridor.^2,4^ We present a patient with a medullary cavernous malformation who had three hemorrhages and neurologic worsening. The surgical approach was based on detailed preoperative imaging. We performed a far lateral posterior fossa exposure to resect the lesion. The details of surgical planning and the microsurgery are presented.

The video can be found here: https://youtu.be/2y-OJ22Zjw8.

## Transcript

### 0:20 Introduction

In this report we describe the microsurgical resection of a medullary cavernous malformation using a far lateral skull base approach.

### 0:27 Patient clinical history

The patient was a 51-year-old retired firefighter without past medical history. He initially had an episode of dizziness, trouble swallowing, which led to the diagnosis of a 9 × 15–mm cavernous malformation with hemorrhage, in the medulla oblongata. He was initially managed conservatively, but subsequently had two more discrete episodes of neurologic worsening. The patient presented to our institution as a referral due to acute worsening of symptoms and follow-up imaging revealing enlargement of the lesion. Due to repeated crescendo hemorrhages, surgical resection was recommended.

Preoperatively, the patient had right leg weakness with poor proprioception in the right lower extremity and numbness in the right upper extremity at the level of the shoulder, and a right sixth nerve palsy.

### 1:11 Imaging studies

Imaging prior to surgery demonstrated a functional MRI which showed a lesion centered in the medulla oblongata above the level of the foramen magnum. There was a small but prominent venous anomaly crossing below the lesion associated with the cavernous malformation. MR tractography demonstrated sensory and corticospinal tracts, which had been pushed laterally to the cavernous malformation by the mass effect. There was a small area of the malformation that was within 2 mm of the posterolateral aspect of the medulla, allowing the possibility of a surgical corridor to the lesion.

This MRI in its sagittal reconstruction shows the lesion in the medulla, just above the foramen magnum. The coronal reconstruction shows an important image in that the lesion is within 2 mm of the left side of the medulla. It also shows its relationship to the vertebral artery, which becomes important at the time of surgical exposure.

MR tractography demonstrates the fibers to be pushed laterally and posteriorly by the lesion itself allowing for a corridor to the malformation. In this coronal image, the left side of the head is on the left side of the image projected. You can see the lesion coming in close proximity to the edge of the medulla, and the measurement demonstrates 1.6 mm. It also demonstrates the relationship of the lesion with the vertebral artery. We predicted what the lengths of working distance would be from the far lateral approach. As shown in this slide, there is approximately 5 cm of reach to the edge of the lesion from the skull, and almost 65 mm from the skin.

### 2:43 Surgical plan

To undertake the procedure, we utilized a far lateral approach from the left side to reach the medulla oblongata. We used lumbar spinal drain, stereotactic guidance, and during the procedure we monitored somatosensory evoked potentials and motor evoked potentials throughout the operation.

### 2:57 Surgical alternative and decision rationale

We consider other surgical approaches such as a posterior approach; however, this risked transgression of significant amount of neural brainstem tissue and, therefore, we settled on a far lateral approach, which was the most direct approach through the minimal amount of brainstem to the lesion. The patient was positioned in the lateral position with the right side down and the head was held rigid within 3-point fixation.

### 3:18 Patient positioning

The patient was positioned in the lateral position and stereotactic guidance was used. We confirmed peducials and planned the incision as a slightly curved incision starting behind the ear, projected medially over the C1 region. We also used neuromonitoring throughout the procedure.

### 3:35 Approach

Here you can see creating the craniotomy with a far lateral approach. We used a cutting bit on the drill to create a small craniotomy which could easily be replaced at the finish of the procedure. Here the dura is exposed and nicely relaxed and we were able to open it with Metzenbaum scissors and then reflect the dura laterally and hold it open using Nurolon sutures. Small brain retractors were used to gently elevate and retract cerebellum medially to give us access to the cistern above the vertebral artery to reach the lesion.

### 4:05 Microsurgical anatomy

Here the retractor is in place and one can see the lower cranial nerves across the screen. We are working below the lower cranial nerves and visualized below the black arrow, the vertebral artery. Brainstem is coming into view and vertebral artery is labeled as V.A.

### 4:22 Medullary incision site

We knew that the lesion laid just adjacent to the vertebral artery based on preoperative planning. As we began to expose the brainstem, we can see the lower cranial nerves entering the medulla. By working these nerves free from their origin, we were able to identify an area on the medulla oblongata with hemosiderin staining. By gently moving the vessels we were able to expose an area on the brainstem, shown with a red circle, where we planned our incision into the brainstem. Here we are incising into the brainstem using an arachnoid knife to carefully open a 2- to 4-mm-length incision. We used dissectors as shown to gently work our way down to the malformation, traversing approximately 1.5 mm to 2 mm of brainstem tissue.

### 5:08 Delineation of malformation

The video shows the point where we encountered hemosiderin staining within the brainstem and the edge of the lesion itself. We did not used any fixed retractors, but rather a continuous motion with small retraction blades to reach the lesion.

### 5:22 Debulking

We now used 0.5-mm irrigating bipolar tip set at a low current and a 1-mm canal knife to gently dissect the lesion free of surrounding tissue. Here one can see the hemosiderin-stained hematoma cavity which we carefully circumnavigated with the canal knife, and we were able to remove in piecemeal fashion the lesion itself, as well as the hematoma cavity. The microscope was moved continuously around the opening in the brainstem to use it as a focal point, and by circumferentially removing the lesion, we carefully moved the microscope as we worked to visualize the edges of the lesion.

### 6:00 Associated developmental venous anomaly

Here is the venous anomaly that was visualized in the preoperative studies to carefully preserve as we removed the lesion itself. We took great care to coagulate the vessels to try to shrink the lesion so we could remove it through the small opening in the brainstem surface. Here you can see the residual of the lesion itself. The associated developmental venous anomaly was left intact after the final resection.

### 6:24 Closure

We were able to obtain a watertight closure using a pericranial flap and replaced the bone without difficulties. Skin incision was closed in layers.

### 6:33 Postoperative scan

Shown on the video is the immediate postoperative scan demonstrating the area of resection of the lesion.

### 6:40 Inpatient course

Postoperatively, the patient was awakened in the operating room and was extubated. In the recovery room, the patient had a weak left foot and reduced sensation in his lower leg, particularly on the right. His eye movements were full and his gag reflex was intact. On the first postoperative day, the patient had a decline in the respiratory status and a reduction in oxygen saturation requiring intubation. A chest x-ray showed a right, middle, and upper lobe lung collapse. Bronchoscopy was performed with the mucus plugs removed which resulted in reinflation of the collapsed lung lobes. The patient was ultimately extubated in the 10th postoperative day; he had gradual clinical improvement until postoperative day 24, when oxygen saturation dropped. Lower extremity ultrasound and chest CT revealed lower extremity DVT and pulmonary emboli. Anticoagulation was initiated and a vena cava filter was placed. This chest CT demonstrates the pulmonary emboli. The patient was eventually weaned off respiratory support and was discharged in the 35th postoperative day to a rehabilitation center.

### 7:38 Follow-up

On the fourth week of follow-up the patient was in rehab with 4/5 strength in the left lower extremity and proprioceptive deficit in the right lower extremity. By 8 weeks of follow-up patient was ambulating with a walker, and with a strength which was 4/5 in both lower extremities, with diminished proprioception in the right lower extremity, which was his biggest obstacle to free ambulation without a walker.
